# Epidemiology, risk factors, and awareness of mycetoma among residents in Eastern Sinnar locality, Sudan, 2021

**DOI:** 10.7189/jogh.15.04005

**Published:** 2025-01-10

**Authors:** Mogahid Gadallh A Abdallh, Sahar Hemeda, Mohammed Elmadani, Bashir Ibrahim, Abd Elbasit Elawad Ahmed

**Affiliations:** 1 Department of Environmental Health and Food Control, Federal Ministry of Health, Khartoum, Sudan; 2 Department of Food Hygiene and Safety, Faculty of Public and Environmental Health, University of Khartoum, Khartoum, Sudan; 3 Department of Epidemiology, Faculty of Public Health, University of El Imam El Mahdi, Kosti, Sudan; 4 Department of Epidemiology, Faculty of Public and Environmental Health, University of Khartoum, Khartoum, Sudan; 5 Department of Health Education, Faculty of Public and Environmental Health, University of Khartoum, Khartoum, Sudan

## Abstract

**Background:**

Mycetoma is a chronic granulomatous disease affecting the skin, subcutaneous tissues, and bones, particularly in tropical and subtropical regions. Sudan, especially its Eastern Sinnar locality, experiences a significant burden due to environmental conditions and limited access to healthcare, while the population's lack of awareness and understanding often leads to delays in diagnosis and treatment.

**Methods:**

We conducted a descriptive cross-sectional, community-based study in Eastern Sinnar, Sudan, to investigate the prevalence, risk factors, and awareness of mycetoma among local residents. Using Cochran’s formula, we calculated a required sample size of 400 participants from a total population of 245 201. Then, we randomly selected these participants from five villages chosen through stratified sampling. Data were collected via a validated questionnaire assessing sociodemographic characteristics and mycetoma-related information, a review of medical records to confirm infection types and prevalence, and interviews with the Directorate of the Mycetoma Centre in Sinnar. We used χ^2^ tests for associations in our analysis, with *P*-values ≤0.05 indicating statistical significance.

**Results:**

The mycetoma prevalence was 5.4%, with males comprising 76.4% of infected cases. However, the difference in infection rates between genders was not statistically significant (*P* = 0.248). While infection rates were higher among certain occupational groups, such as farmers and shepherds, the association between occupation and mycetoma infection was non-significant (*P* = 0.107). We also found no significant associations with educational level (*P* = 0.104) or age (*P* = 0.514), but did detect significant associations for family history of infection (*P* < 0.001), animal ownership (*P* = 0.004), and not wearing shoes during work (*P* = 0.05). Awareness of mycetoma was relatively high, with 78.3% of respondents acknowledging the disease, though knowledge gaps remained, especially regarding its transmission, with only 36.1% believing it to be transmissible.

**Conclusions:**

The study highlights the need for targeted health education programmes, particularly emphasising protective footwear and safe animal-handling practices. These findings are crucial for informing public health strategies aimed at reducing the burden of mycetoma in endemic regions such as Eastern Sinnar.

Mycetoma, a chronic granulomatous disease characterised by localised infection of the skin, subcutaneous tissue, and bones, remains a significant, yet neglected health issue, predominantly affecting individuals in tropical and subtropical regions [1–6]. It is caused by various species of bacteria and fungi, with infection pathways often linked to minor trauma or wounds that encounter contaminated soil or plant material [7,8]. The disease, marked by the formation of tumefactions, sinuses, and discharge containing grains, can lead to severe deformities, disability, and social stigmatisation if left untreated [8].

It is recognised as a neglected tropical disease by the World Health Organization (WHO). Globally, it is estimated that around 10 000 new cases are reported annually, but due to underreporting, the actual figure is believed to be significantly higher. The global burden of mycetoma is largely concentrated in regions like Sudan, Mexico, and parts of Africa, Asia, and South America [4]. In Sudan specifically, mycetoma cases have reached endemic proportions, with a prevalence rate of 14.5 per 100 000 people [9], making the country one of the global hotspots for the disease [10]. The endemic nature of mycetoma in Sudan is attributed to several factors, including the hot and arid climate, agricultural practices, and socioeconomic conditions that expose the population to the causative agents [11–12]. Regions such as the Eastern Sinnar locality are disproportionately affected due to their environmental conditions and limited access to healthcare facilities [13]. Mycetoma is often referred to locally as ‘Madura foot’, a term derived from the Madura region in India where the disease was first described [14,15].

The burden of mycetoma in Sudan is exacerbated by a lack of awareness and understanding of the disease among the general population [16]. Many residents in endemic areas attribute the symptoms to other conditions or traditional beliefs, delaying the seeking of appropriate medical care [16]. This delay in diagnosis and treatment often leads to advanced stages of the disease, where more aggressive and invasive interventions are required, resulting in higher morbidity and economic burden on affected individuals and their families [11,17].

In terms of public health response, Sudan has made an effort to address mycetoma through initiatives such as the establishment of the Mycetoma Research Center in Khartoum, which serves as a hub for research, training, and treatment of the disease [12]. However, significant challenges remain despite these efforts. The limited availability of diagnostic tools and treatment options and the lack of trained healthcare professionals in rural and remote areas currently hampers effective disease management [12].

Socioeconomic and environmental conditions of Eastern Sinnar contribute to the disease's high prevalence. The region's hot, arid climate and predominantly agricultural economy create an environment conducive to exposure to mycetoma's causative agents, such as soil-dwelling fungi and bacteria. Frequent contact with contaminated soil and plants, through activities like farming and shepherding, increases infection rates among residents. Additionally, limited access to healthcare services in rural areas leads to delayed diagnosis and treatment. Socioeconomic challenges, including low income and education levels, exacerbate the situation by reducing awareness and preventive measures. These factors, coupled with the spatial distribution of mycetoma cases in Eastern Sinnar evidenced by a study of 594 confirmed patients from 1991 to 2020 at the Mycetoma Research Centre in Khartoum, underscore the critical need for targeted research and intervention in this highly endemic region [18].

In Sinnar, Sudan, the treatment of mycetoma involves a combination of antimicrobial and antifungal drugs tailored to the type of infection. For actinomycetoma, the primary regimen includes Amikacin sulfate (15 mg/kg) combined with Co-trimoxazole (14 mg/kg twice daily). In cases of resistance, alternatives such as streptomycin sulfate with co-trimoxazole or rifampicin are used. Treatment generally extends beyond a year and is crucial both pre- and post-operatively to support surgical interventions and reduce relapse, with a cure rate between 60% and 90%. Eumycetoma is managed with antifungals such as ketoconazole (400–800 mg daily) or itraconazole (400 mg daily), with treatment duration varying from months to years and regular liver function monitoring due to potential drug effects. Surgical approaches focus on complete removal or debulking of the lesion, with care to avoid rupturing capsules or leaving residual fungal elements and may include post-operative iodine solutions to prevent recurrence. In advanced cases, amputation might be necessary, but less invasive procedures are preferred when feasible to minimise social impacts [19].

Understanding the knowledge and attitudes of residents in endemic areas is crucial for the effective management and prevention of mycetoma. Public awareness and attitudes towards the disease influence health-seeking behaviour, compliance with treatment regimens, and overall disease outcomes. Moreover, community knowledge can drive or hinder the implementation of preventative measures and early detection initiatives [20,21].

In this study, we aimed to assess the prevalence, risk factors, and awareness of mycetoma among residents of the Eastern Sinnar locality in Sudan, or more specifically, to evaluate community knowledge and attitudes towards mycetoma, identify gaps in understanding, and investigate key factors influencing the risk of infection.

## METHODS

We conducted this descriptive cross-sectional community-based study in the Eastern Sinnar locality, Sinnar, Sudan, to investigate residents' prevalence, risk factors, and awareness of mycetoma. Our sample included 400 participants, determined using Cochran's formula [22]:



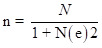



Data collection involved three distinct methods to ensure a comprehensive understanding of mycetoma within the community. First, we administered a pre-prepared and validated questionnaire to residents to gather information on their sociodemographic characteristics and various aspects related to mycetoma. The questionnaire was either self-administered by literate participants or, for illiterate individuals, with the assistance of literate family members or trained data collectors. We then reviewed patients' medical records to identify the types of infections and confirm the self-reported prevalence of mycetoma. Lastly, we conducted interviews with the Directorate of the Mycetoma Centre in Sinnar to gain detailed insights into various aspects of mycetoma, ensuring a well-rounded understanding of the disease within the community.

We used a stratified sampling method to ensure that the sample was representative of the population at multiple levels. The process began by defining the population of Sinnar State as the study population, with the state divided into seven localities, each considered a separate stratum. We then randomly selected the Eastern Sinnar locality from these seven strata.

In the second stage, we randomly chose the Wad Unsa administrative unit from the four administrative units in Eastern Sinnar. Out of the 38 villages in Wad Unsa, we further randomly picked five villages: Elragal Ibrahim, Wad Hassan, Elkananab, Eridiba, and Mahala 22. The total population of these five villages was 2531, distributed as follows: 439 in Elragal Ibrahim, 749 in Wad Hassan, 766 in Elkananab, 355 in Eridiba, and 222 in Mahala 22.

We then gathered the required sample size of 400 participants from the total population of 2531 individuals in Wad Unsa by proportionally allocating it across the five selected villages, with 76 individuals coming from Elragal Ibrahim, 116 from Wad Hassan, 119 from Elkananab, 55 from Eridiba, and 34 from Mahala 22. Finally, we used simple random sampling within each village to select participants, ensuring that every resident had an equal chance of being included in the study.

We performed the data analysis in SPSS, version 24.0 (IBM, Armonk, New York, USA). Associations between variables were evaluated using the χ^2^ test, with a significance level set at *P* ≤ 0.05.

We received ethical clearance from the Council of Medical and Health Studies, the Ministry of Health, and the Mycetoma Centre in Sinnar. Informed consent was obtained from all participants.

## RESULTS

### Sociodemographic characteristics

The population in the Eastern Sinnar locality was predominantly young, with 39.5% of residents aged between 18–31 years. The low percentage (17%) of individuals over 45 years old may have reflected high mortality rates, migration patterns, the impact of the coronavirus disease 2019 (COVID-19) pandemic, or other socioeconomic factors. The unusual age distribution in the population was likely due to several factors, including the emigration of younger adults and lower life expectancy due to limited access to healthcare. We observed a notable gender disparity, with males representing 63.2% of the population and females 36.8%. Educational attainment varied widely: 34.8% had a basic/elementary education and 26.5% reached university and postgraduate levels. However, 15% of the population was illiterate, indicating room for improvement in educational outreach. Socially, the majority (73.3%) were married, reflecting strong marital trends within the community. Regarding occupation, a third of individuals (33.5%) were engaged in unspecified jobs, 24.5% in farming, and 19.8% were employed, suggesting a diversified economy with a significant agricultural component. Income levels also show disparity, with 60.8% earning more than SDG 2000-monthly, while 10.3% earn less than SDG 750. Most residents (99.3%) were originally from Sinnar State, indicating low migration from other states (**Table 1**).

**Table 1:** Demographic and socio-economic characteristics of study participants

Characteristics	n (%)
Age distribution	
*<18*	72 (18.0)
*18–31*	158 (39.5)
*32–45*	102 (25.5)
*>45*	68 (17)
Sex distribution	
*Male*	253 (63.2)
*Female*	147 (36.8)
Educational level	
*Illiterate*	60 (15.0)
*Basic/elementary*	139 (34.8)
*Secondary*	87 (21.8)
*University/postgraduate*	106 (26.5)
Khalwa	8 (2.0)
Social status	
*Married*	293 (73.3)
*Single*	74 (18.5)
*Divorced*	20 (5.0)
*Widowed*	13 (3.2)
Occupational status	
*Farmer*	98 (24.5)
*Laborer*	62 (15.5)
*Employee*	79 (19.8)
*Shepherd*	27 (6.8)
*Others*	134 (33.5)
Monthly income in SDG	
*<750*	41 (10.3)
*750–1000*	35 (8.7)
*1000–1500*	53 (13.2)
*1500–2000*	28 (7.0)
*>2000*	243 (60.8)
Original residence	
*Sinnar state*	397 (99.3)
*Other state*	3 (0.7)

### Mycetoma knowledge and awareness

Awareness of mycetoma was high among residents, with 78.3% acknowledging the disease. Among those aware, 39.6% correctly identify mycetoma as an inflammatory disease. However, only 36.1% knew it to be transmissible, indicating a need for better education on transmission mechanisms. Most residents (54.6%) recognised disability as a major complication of the disease, underscoring its severe impact. Knowledge of prevention was also substantial, with 74.1% understanding prevention methods, primarily through health education and wearing shoes (**Table 2**).

**Table 2:** Knowledge and awareness of mycetoma among study participants

Knowledge area	n (%)
Awareness of mycetoma	
*Yes*	313 (78.3)
*No*	87 (21.7)
Definition of mycetoma	
*Inflammatory disease*	124 (39.6)
*Tumor*	94 (30.0)
*I don't know*	95 (30.4)
Mode of transmission	
*Yes*	113 (36.1)
*No*	200 (63.9)
Complications of mycetoma	
*Disability*	171 (54.6)
*Amputation*	45 (14.4)
*Death*	7 (2.2)
*All*	90 (28.8)
Prevention knowledge	
*Yes*	232 (74.1)
*No*	81 (25.9)

### Infection and practices

Mycetoma infection rates were relatively low, with only 5.4% of respondents reporting an infection. Among the infected participants, 64.7% had bacterial infections, 17.6% had fungal infections, and 17.6% experienced mixed infections. The duration of infection was less than one year for 64.7% of the cases, while 35.3% had been infected for over a year. In terms of symptoms, 47.1% exhibited a granulomatous appearance, 29.4% experienced swelling, and 23.5% had ulceration. Regarding responses to the infection, 82.3% sought medical help, 11.8% consulted a traditional healer, and 5.9% took no action. Personal hygiene practices were reported by 89% of participants, while 11% did not follow proper hygiene. Notably, 58.3% of participants did not wear shoes during work, and among those who did, 76.6% wore shoes that did not cover their feet, with only 23.4% using fully covering footwear (**Table 3**).

**Table 3:** Infection rates and associated behavioural practices

Infection and practices area	n (%)
Mycetoma infection	
*Yes*	21 (5.4)
*No*	379 (94.6)
Types of infection	
*Bacterial*	11 (64.7)
*Fungal*	3 (17.6)
*Mixed*	3 (17.6)
Duration of infection	
*Less than one year*	11 (64.7)
*More than one year*	6 (35.3)
Signs of infection	
*Granulomatous appearance*	8 (47.1)
*Swelling*	5 (29.4)
*Ulceration*	4 (23.5)
Response to infection	
*Seek medical help*	
*Traditional healer*	2 (11.8)
*No action*	1 (5.9)
Personal hygiene practices	
*Yes*	356 (89.0)
*No*	44 (11.0)
Wearing shoes during work	
*Yes*	167 (41.7)
*No*	233 (58.3)
Type of shoes	
*Covering the feet*	39 (23.4)
*Not covering the feet*	128 (76.6)

### Risk factors for mycetoma infection

Farmers and shepherds, despite being a smaller portion of the population, were more frequently infected, suggesting occupational exposure is a significant risk factor. Infection rates were spread across all educational levels, indicating that education alone is not a protective factor. Age did not significantly correlate with infection rates (*P* = 0.514), and men were slightly more affected than women with a non-significant difference (*P* = 0.248), aligning with their higher presence in the population. A significant association existed between mycetoma infection and family history (*P* = 0.000), pointing to potential genetic or environmental factors. Owning animals, with goats as predominant species, as well as cows, sheep, and donkeys, significantly increased the risk of infection (*P* = 0.004), suggesting animal contact as a potential risk factor. Not wearing shoes during work was also significantly associated with higher infection rates (*P* = 0.05), underscoring the importance of protective footwear in preventing mycetoma (**Table 4**).

**Table 4:** Analysis of risk factors about mycetoma infection

Risk factor	Infected, n (%)	Not infected, n (%)	Total, n (%)	*P*-value
Occupation				0.107
*Laborer*	5 (1.25)	57 (14.25)	62 (15.5)	
*Employee*	1 (0.25)	78 (19.5)	79 (19.75)	
*Farmer*	4 (1.0)	94 (23.5)	98 (24.5)	
*Shepherd*	3 (0.75)	24 (6.0)	27 (6.75)	
*Others*	4 (1.0)	130 (32.5)	134 (33.5)	
Educational level				0.104
*Illiterate*	2 (0.5)	58 (14.5)	60 (15.0)	
*Islamic school*	3 (0.75)	17 (4.25)	20 (5.0)	
*Primary school*	8 (2.0)	147 (36.75)	155 (38.75)	
*Secondary*	3 (0.75)	56 (14.0)	59 (14.75)	
*University*	1 (0.25)	105 (26.25)	106 (26.5)	
Age				0.514
*<18*	1 (0.25)	71 (17.75)	72 (18.0)	
*18–31*	9 (2.25)	149 (37.25)	158 (39.5)	
*32–45*	4 (1.0)	98 (24.5)	102 (25.5)	
*>45*	3 (0.75)	65 (16.25)	68 (17.0)	
Sex				0.248
*Male*	13 (3.25)	240 (60.0)	253 (63.25)	
*Female*	4 (1.0)	143 (35.75)	147 (36.75)	
Family infection history				0
*Yes*	8 (2.6)	15 (4.7)	23 (7.3)	
*No*	9 (2.9)	281 (89.6)	290 (92.7)	
Owning animals				0.004
*Yes*	15 (3.75)	200 (50.0)	215 (53.75)	
*No*	2 (0.5)	185 (46.25)	187 (46.75)	
Wearing shoes during work				0.05
*Yes*	5 (2.1)	162 (38.3)	167 (40.4)	
*No*	12 (2.6)	217 (51.6)	229 (54.2)	

## DISCUSSION

Mycetoma is a chronic granulomatous disease primarily affecting individuals in tropical and subtropical regions. This neglected health issue is particularly significant in countries like Sudan, where environmental and socioeconomic factors contribute to its prevalence. This descriptive cross-sectional community-based study in the Eastern Sinnar locality aimed to investigate the prevalence and risk factors of mycetoma among residents, providing valuable insights for targeted interventions.

We observed a mycetoma prevalence of 5.4%, aligning with the findings of a previous study which reported 44 cases [23] and another which reported a prevalence of 1.5% [24]. This prevalence is higher compared to a study conducted by Hassan et al. [25] in the same locality, who reported a prevalence of 0.87%, as well as other studies which found a prevalence of 14.5 per 1000 inhabitants (1.45%) [16,26]. The prevalence was higher among males (63.2%) compared to females (36.8%), and most common among individuals aged 18–31 years (39.5%). These results are consistent with [26], who found a median age of 23 years, with 60% of cases in males and 40% in females. Mycetoma predominantly affects disadvantaged populations in sub-Saharan Africa, with a notable concentration in Sudan, where recent surveys revealed higher prevalence rates than had previously been estimated [27]. In West Africa, approximately 2685 cases have been documented from 1929 to 2020, with Senegal alone accounting for 74.1% of these cases [28]. In Egypt, a scoping review highlighted a higher incidence of mycetoma than reported previously, emphasising the need for further research to accurately assess its prevalence [29].

The disease tends to be more prevalent in households lacking adequate water, sanitation, and electricity and is frequently undertreated due to restricted healthcare access and high costs associated with treatment [30]. The spatial distribution of mycetoma has been associated with specific soil types, especially light clay soils, and the nature of the land cover [18]. A systematic review by van den Sande et al. [9] identified Sudan as one of the countries with the highest mycetoma cases, alongside India and Mexico.

Our findings showed that the incidence of mycetoma in Sudan is significantly shaped by sociodemographic factors, consistent with previous research. Younger individuals, particularly those aged 11–30 years, were more frequently affected, aligning with studies that report 64% of mycetoma cases occur within this age group [31]. Males were disproportionately represented, accounting for 56.6% to 79.6% of cases [31,32], which may reflect gender-specific occupational exposure. Indeed, our data aligns with the established correlation between mycetoma and agricultural occupations, as 62.1% to 69.7% of cases are linked to farming activities, likely due to increased contact with soil-borne pathogens [30,31]. We further identified farmers as being at higher risk due to their occupational exposure to contaminated soil and plant material, which are common sources of the bacteria and fungi that cause mycetoma. The nature of agricultural work, often done barefoot, increases the likelihood of sustaining minor injuries that serve as entry points for infection. In addition, individuals with lower education levels may lack knowledge or awareness about the disease and its transmission, making them less likely to adopt preventive measures such as wearing protective footwear. Limited health literacy may also contribute to delays in seeking medical care, leading to more advanced stages of the disease.

Despite high awareness of mycetoma (78.3%), significant gaps persist in understanding its nature, with only 39.6% identifying it as an inflammatory disease and 36.1% believing it to be transmissible. These knowledge gaps, combined with barriers to healthcare access [30], underscore the need for targeted health education, especially among agricultural communities, as 52.9% of respondents believed that educational interventions could achieve prevention. Furthermore, the recognition of disability as a major complication by 54.8% of respondents highlights the severe social and physical consequences of the disease. Thus, while sociodemographic factors, particularly age, gender, and occupation, are key determinants of mycetoma incidence, addressing educational and healthcare access barriers is crucial for better disease management.

Most of those infected sought medical help, demonstrating a reliance on professional healthcare services. However, a significant number of residents did not wear shoes during work (58.3%) and many wore inadequate footwear (76.6%), highlighting a critical area for intervention. Promoting the use of protective footwear could significantly reduce infection rates. Occupational exposure, particularly among farmers and shepherds, is a significant risk factor for mycetoma infection. The association between mycetoma and owning animals suggests that animal contact is a potential transmission route. Not wearing shoes during work is significantly associated with higher infection rates, emphasising the need for occupational health interventions, such as promoting protective footwear and safe animal handling practices. We found that many individuals did not wear protective footwear, a key preventive measure against mycetoma. This could be due to financial barriers, as proper shoes may be unaffordable for many residents, or due to cultural practices or norms. Additionally, there may be a lack of awareness regarding the protective benefits of footwear in preventing mycetoma. Addressing these barriers requires public health interventions such as the provision of affordable footwear and educational campaigns to promote the importance of wearing shoes, especially in high-risk occupational settings.

Understanding community knowledge and attitudes is crucial for effective mycetoma management. While general awareness is high, we found critical gaps in knowledge about transmission and prevention. Many residents adhere to good hygiene practices, but the low use of protective footwear during agricultural activities presents a significant risk. Educational programmes should focus on improving knowledge about the disease, its transmission, and effective prevention measures. To address the identified gaps and improve mycetoma management in Eastern Sinnar, several public health interventions are recommended. These include focussing on young adults and men who constitute a significant portion of the population to enhance their understanding of mycetoma; developing and implementing educational programmes to improve knowledge about mycetoma transmission and prevention, particularly among farmers and other high-risk groups; promoting the use of protective footwear and safe animal handling practices to reduce occupational exposure to mycetoma; enhancing access to healthcare services, particularly in rural areas, to ensure timely diagnosis and treatment of mycetoma; and continuing to promote personal hygiene practices to prevent infections and reduce mycetoma incidence.

Additionally, the socioeconomic factors influencing mycetoma progression further underscore the need for targeted interventions in these communities. Patients often present at advanced stages due to inadequate healthcare access, leading to severe morbidity and, in some cases, amputation, particularly in rural areas where treatment is limited [33]. The economic burden of managing mycetoma-related disabilities exacerbates poverty within these communities, as the disease often requires costly interventions [34]. Challenges in accessing early treatment and the high cost of care contribute to higher amputation rates, particularly in eumycetoma cases, highlighting the importance of reducing delays in care [35]. Addressing these socioeconomic barriers, including promoting healthcare access and increasing awareness, is critical to improving disease outcomes [36]. Expanding on the complications identified in the study, untreated mycetoma can lead to significant disability, which has severe social and economic consequences. In rural areas like Eastern Sinnar, where agriculture is the main source of livelihood, disability can result in an inability to work, leading to income loss and further economic hardship. Additionally, social stigma related to the visible deformities caused by advanced mycetoma can lead to isolation, discrimination, and reduced quality of life. Discussing these broader impacts underscores the importance of timely diagnosis and treatment to prevent long-term disability and its ripple effects on affected individuals and their families.

### Implications for future research

Future research efforts should focus on large-scale longitudinal studies to determine the true prevalence and incidence rates and investigate environmental and climatic factors that influence mycetoma distribution. Understanding socioeconomic and cultural factors, such as occupational risks and traditional practices, is also crucial to developing effective prevention strategies. Further exploration of the relationship between animal ownership and mycetoma infection is likewise needed, as this may reveal zoonotic aspects of the disease.

Developing and testing targeted health education interventions that increase awareness and change behaviours, particularly in rural areas, is another crucial step in this sense. Barriers to timely diagnosis and treatment should also be explored, and clinical trials are needed to assess the efficacy of both current and novel treatment protocols. Additionally, community-based interventions that promote the use of protective footwear and safe animal-handling practices should be evaluated, alongside the impact of improved sanitation and hygiene practices. Genetic and molecular studies are required to better understand host susceptibility, pathogen diversity, and immune response. Finally, economic assessments of mycetoma’s burden and the cost-effectiveness of various prevention and treatment strategies will provide critical insights for public health policy and resource allocation. Enhanced community engagement and the development of surveillance systems will aid in early detection and management, improving overall outcomes.

Evaluating the economic burden of mycetoma and the cost-effectiveness of various prevention and treatment strategies will inform public health policy and resource allocation. Lastly, the role of community engagement in the success of mycetoma prevention and control programmes should be studied, and enhanced community-based surveillance and reporting systems should be investigated for early detection and management of mycetoma cases.

### Study limitations

The study has several limitations that should be considered. First, as it adopted a cross-sectional design, it captured only a snapshot in time, limiting the ability to establish causality or observe changes over time. Our reliance on self-reported data may have introduced recall bias or inaccuracies in reporting mycetoma symptoms and infection history. The study sample, while representative of the Eastern Sinnar locality, may not be generalisable to other regions with different environmental, socioeconomic, or cultural contexts. The relatively small sample size might limit the statistical power to detect significant associations between variables. Additionally, our focus on visible signs of mycetoma and awareness may have overlooked asymptomatic cases or those with milder symptoms, leading to an underestimation of the true prevalence. Limited access to diagnostic tools and healthcare facilities in rural areas could have further skewed the results, as individuals with undiagnosed mycetoma might not have been included. Finally, we did not account for potential confounding factors such as other health conditions, nutritional status, or genetic predispositions, which could have influence mycetoma susceptibility and outcomes.

## CONCLUSIONS

This study underscores the urgent need for targeted public health interventions to address the high prevalence of mycetoma in Eastern Sinnar. Farmers, labourers, and other high-risk occupational groups are particularly vulnerable due to their frequent exposure to contaminated soil and lack of protective footwear. While awareness of mycetoma is relatively high, significant gaps in understanding its transmission and prevention persist. Practical interventions are essential to reducing the its burden. Health education programmes specifically tailored for farmers and rural communities should emphasise the importance of wearing protective footwear, recognising early symptoms, and seeking timely medical care. These programmes could be delivered through community health workers or school-based initiatives, ensuring greater reach. Integrating mycetoma education into existing public health campaigns would maximise impact. An equally important intervention is the provision of affordable protective footwear. Policymakers could implement subsidy programmes or collaborate with non-governmental organisations to ensure vulnerable populations have access to culturally appropriate footwear for agricultural work. The findings from this study have broader implications beyond Eastern Sinnar. Many regions in Sudan and other African countries share similar socio-economic and environmental conditions, making them equally susceptible to mycetoma. The strategies proposed here – focussed on education, prevention, and improved healthcare access – can be applied in other endemic regions. Cross-regional collaboration and sharing of best practices will enhance the effectiveness of mycetoma control efforts across Africa.
